# Health Implications of Adults' Eating at and Living near Fast Food or Quick Service Restaurants

**DOI:** 10.1038/nutd.2015.18

**Published:** 2015-07-20

**Authors:** J Jiao, A V Moudon, S Y Kim, P M Hurvitz, A Drewnowski

**Affiliations:** 1 Graduate Program in Community and Regional Planning, School of Architecture, University of Texas at Austin, Austin, TX, USA; 2 Department of Urban Design and Planning, College of Built Environments, University of Washington, Seattle, WA, USA; 3 Department of Environmental and Occupational Health Sciences, School of Public Health, University of Washington, Seattle, WA, USA; 4 Department of Epidemiology, School of Public Health, University of Washington, Seattle, WA, USA

## Abstract

**Background:**

This paper examined whether the reported health impacts of frequent eating at a fast food or quick service restaurant on health were related to having such a restaurant near home.

**Methods:**

Logistic regressions estimated associations between frequent fast food or quick service restaurant use and health status, being overweight or obese, having a cardiovascular disease or diabetes, as binary health outcomes. In all, 2001 participants in the 2008–2009 Seattle Obesity Study survey were included in the analyses.

**Results:**

Results showed eating ⩾2 times a week at a fast food or quick service restaurant was associated with perceived poor health status, overweight and obese. However, living close to such restaurants was not related to negative health outcomes.

**Conclusions:**

Frequent eating at a fast food or quick service restaurant was associated with perceived poor health status and higher body mass index, but living close to such facilities was not.

## Introduction

The rise of obesity has been explained by dietary changes and parallel increases in the supply and consumption of high-energy but low-nutrient foods. Over the past three decades, trends show increases in eating away from home and energy gained from sugars and fats.^1, 2^ In a longitudinal prospective study, out-of-home eating, including at fast food restaurants, was associated with weight status and multiple metabolic outcomes.^3^ Eating at fast food restaurants was found to relate to poorer diets in the general population,^4^ in a group of pregnant women,^5^ and to higher rates of obesity in rural areas of the Midwest.^6^ However, the health effects of eating out frequently might differ for men and women.^7^

Poor diets and health may be due to increases in the number of fast food and other pay-before-you-eat establishments, which offer a restricted choice of healthy out-of-home foods.^8^ Hence public health professionals have hypothesized that increased exposure to the now ubiquitous restaurants might be the mechanism through which health and diets might be impacted. In response, a large literature investigated how proximity and access to fast food restaurants relate to health. Examining associations between home-based distances to or density of restaurants near homes and a variety of health outcomes, this literature produced mixed results. A review of 40 articles found that access to fast food restaurants was related to higher body mass index (BMI) in six studies, and not related in four studies.^9^ Another review found some evidence that residents with limited access to fast foods had healthier diets and lower levels of obesity.^10^ The local food environment was associated with recommended dietary intake^11^ and with the prevalence of obesity.^12^ One study found that multiple food options in a neighborhood decreased the risk of being obese.^13^ Obesity prevalence was linked to living in states or counties with higher density of fast food restaurants;^14^ in census units with more fast food restaurants or convenience stores;^15^ and in areas with higher concentrations of local restaurants.^16^ No such associations were found in other studies.^17, 18, 19^ One study found lower BMI and lower prevalence of obesity to be associated with living in an area with higher densities of stores offering a choice of healthy foods, but not with higher densities of restaurants.^20^ Weight gain was higher in older adults living nearer fast food restaurants.^21^ And while no association was found with cardiovascular mortality,^22^ the risk of having a stroke was shown to increase with the density of fast food restaurants in the home neighborhood.^23^

Disadvantaged groups also appeared more likely to live in neighborhoods with concentrations of unhealthy food sources than their wealthier counterparts.^24, 25^ Of 40 studies reviewed, 12 found fast food restaurants to be more prevalent in areas housing ethnic minority groups.^9^ Another review of 33 studies found 14 studies where the availability of fast food outlets was correlated with higher deprivation; but 13 studies that yielded conflicting results.^26^ In several North American studies, fast food accessibility was correlated with different measures of neighborhood deprivation,^27, 28^ and with the odds of being obese.^29, 30, 31^ No such association was found in another study.^32^ In contrast, low-income residents of rural areas were found to live closer to healthier fares offered in fast food outlets than their higher-income counterparts.^33^

So far, most studies measured exposure as the spatial proximity to food in the home or school neighborhood environment. Few were able to relate to a population's actual consumption pattern, including actual food intake, food purchases or the prevalence of eating out.^1, 7, 11, 17^ Even more importantly, they lacked data on where people actually ate out or purchased food, and specifically whether they ate or purchased food in the neighborhood of exposure. The lack of spatially matched data on exposure and consumption makes it difficult if not impossible to untangle the direct versus the indirect effects of exposure on behavior. One study found that access to outlets with take-away or fast food could not predict the consumption of foods offered in these establishments.^34^ These limitations, plus the fact that most studies remained cross-sectional, and used different, typically untested measurements of environment, might in part explain the mixed results yielded to date.

This study uses self-reported data on eating at and using specific fast food and quick service restaurants. Geocoded participants' home and work addresses and locations of all restaurants served to examine whether using fast food and quick service restaurants and living close to them were associated with reported health status, being overweight or obese, having cardiovascular disease (CVD) or diabetes.

## Materials and methods

### Population

Participants came from the Seattle Obesity Study, a population-based study of social disparities, diet quality and health in King County, WA. A 20-min telephone survey was conducted on a stratified random sample of 2001 randomly selected adult residents of King County, WA. Administered between October 2008 and March 2009, the survey was approved by the Institutional Review Board at University of Washington. Seattle Obesity Study participants were representative of the population in King County in terms of race and ethnicity, income and household size; and similar to the Behavioral Risk Factor Surveillance System 2007 sample in terms of age and gender. The participants' individual demographic and socioeconomic characteristics included age, gender, ethnicity, number of children <12 years, number of children between 12 and 18 years, household size, education, employment and annual household income (Table 1).

### Fast food and quick service restaurant use

The use of fast food or quick service restaurants came from the survey question ‘when you eat out, how often do you go to a quick service restaurant?' where the respondent was told ‘a quick service restaurant is a place where you pay before you eat, such as a fast food restaurant or coffee shop.' Respondents could select one of four answers: (1) never or ⩽2 times a month; (2) 2 to 3 times per month; (3) once a week; (4) 2–3 times per week; and (5) >4 times per week. Following accepted healthy eating standards,^35^ the data were dichotomized at (4), 2–3 times per week, henceforth noted as <2 and ⩾2 a week. Respondents were then asked ‘which quick service restaurant do you go to most often,' and prompted to provide the name of the restaurant. They also gave the names of the principal streets at the closest street intersection of the restaurant, which allowed researchers to locate the restaurant most frequently used relative to those in the County's inventory.

### Fast food and quick service restaurants

The restaurants were extracted from the food permit data provided by the Public Health Seattle and King County, which contained 10 254 records. Fast food restaurants were classified by the University of Washington Urban Form Lab as those of nationally recognized chains that sold meals that had been designed off-site, and were expected to be ready by the time a customer's change is handed over.^36, 37^ Similar to fast food restaurants, quick service establishments did not offer full table service and sold meals that had been designed off-site. They promised a somewhat higher quality of food and atmosphere, but often at higher average prices than charged at fast food restaurants.^38^ Each one of the 606 fast food and 2395 quick service restaurants in the sample had one food permit. Under the quick service category, there were 302 permits identifying bakeries/delis, 786 indicating that ethnic foods were offered, and 1307 offering standard food. Fast food and quick service restaurants constituted 46% of the total inventory of restaurants in King County.^39^

### Outcome variables

The health outcome, general health status, was from the survey question 'Would you say that in general your health is: excellent/very good/good/fair/poor?' The measure was dichotomized into perceived fair/poor health versus otherwise.

Survey questions on weight and height were used to calculate BMI. Participants with a BMI ranging between 25 and 29.99 were classified as being overweight, and those with a BMI of ⩾30 kg/m^2^ were obese.

Having diabetes or CVD was from two survey questions ‘Have you ever been told by a doctor, nurse or another health professional that you have diabetes/any kind of heart disease?' These health outcomes were dichotomized.

### Geocoding

The residential and work addresses of Seattle Obesity Study respondents were geocoded to the centroid of the home or work parcel using the 2008 King County Assessor parcel data. Geocoding followed standard methods in ArcGIS, version 9.3.1 (ESRI, Redlands, CA, USA). Address records that failed the automatic geocoding (30% using a 100% match score) were manually matched using a digital map environment with annotated layers from the reference data augmented by online resources such as GoogleMaps, QwestDEX, and Yelp. Each home and work point was double-checked by a separate technician for plausibility (the parcel being a residential land use) and accuracy (the location being on the correct parcel).

Fast food and quick service restaurants included 3001 food permit records out of the Public Health Seattle and King County 10 254 records. Permit addresses were also geocoded to King County parcel centroids, and using ArcGIS, version 9.3.1 (ESRI); 99.6% of the food permit addresses were geocoded.

### Distance to frequently used and closest restaurant

Distance measures were computed from each respondent's home and work to the fast food and quick service restaurant that respondents reported using and to the same type of restaurant nearest their homes. Network distance was calculated in ArcGIS 9.3.1 and using ESRI StreetMap Premium North America NAVETQ 2009 Release 1 (2008), which determined a route based on driving time impedance. Thus, the distances (in miles) represented the fastest, but not necessarily the shortest route subjects would likely drive from home to the nearest fast food and quick service restaurant along the existing road network.

### Analyses

Logistic regression was used for binary health outcomes: self-reported health, being overweight, being obese, having CVD and having diabetes. Of the four modeling approaches, model 1 estimated the relationship between fast food or quick service use and health outcomes without the adjustment for any covariate. Model 2 added distance from home to the closest restaurant. Model 3 adjusted for demographic characteristics including gender, ethnicity, number of children under 12, number of children between 12 and 18 and household size. Socioeconomic status indicators (for example, income, education and employment) were included in model 4. Models 5–8 investigated the relationship between all the variables in model 4 with being overweight, being obese, having CVD and having diabetes, respectively. Interaction effects were also examined between socioeconomic status and fast food or quick service use on health outcomes after adjusting for demographic characteristics. Analyses were conducted using R, version 3.1 (GNU General Public License).

## Results

### Descriptive analyses

The ⩾2 per week fast food or quick service user population (408) was smaller than that of <2 per week users (1584); nine respondents did not report on fast food or quick service use (Table 1). Median distances to the closest fast food or quick service restaurants for frequent fast food or quick service users were 0.83 and 0.62 miles, respectively. Because the two distance measures were highly correlated (*r*=0.86) and health effect analyses for either distance measure yielded similar results, reported model results only included the measure of distance to the closest quick service restaurant.

Fourteen percent of the participants reported being in perceived poor health; 56% were overweight; 21% were obese; 10% had a CVD; and 9% had diabetes. The majority of the population consisted of women (62%) and Whites (78%). Sixty-one percent were employed; 82% had at least some college education and 60% had an annual income of more than $50,000.

### Analyses

Using a fast food or quick service ⩾2 times per week significantly increased the risk of reporting being in perceived poor health, after adjusting for demographic characteristics and socioeconomic status (Table 2, model 4; odds ratio (OR) 1.61; 95% confidence interval (CI) 1.13–2.28). Increasing the distance to the closest quick service restaurant decreased the risk of reporting perceived poor health, but the association was not present after adjusting for socioeconomic status. Being employed, having higher educational attainment and a higher income decreased the risk of reporting perceived poor health.

Frequent fast food or quick service use significantly increased the risk of being overweight (Table 3, model 5; OR 1.66; 95% CI 1.28–2.16). Increasing age was marginally associated with the risk of being overweight, while being male was a strong predictor. Frequent fast food or quick service use was also associated with the risk of being obese (Table 3, model 6: OR 2.02; 95% CI 1.52–2.69). Having some college education and an income of >$50 000 decreased the risk of being obese. However, age and being male were not related to the risk of being obese. Distance to the closest fast food or quick service restaurant was not associated with being overweight or obese in any of the models.

Finally, using a fast food or quick service restaurant ⩾2 times per week was not associated with having CVD or diabetes (Table 3, model 7 and model 8). Increasing age was marginally associated with having these diseases. Being male was a strong predictor of increased risk of having CVD. Being unemployed also strongly increased the risk of having CVD or diabetes.

No interaction effects were found between fast food or quick service use and socioeconomic status for any of the health outcomes (data not shown). Distance to the closest fast food or quick service restaurant was also not significant in either models.

## Discussion

In line with past studies, the present analyses found that using a fast food restaurant or quick service restaurant ⩾2 times a week was significantly related with being in perceived poor health, overweight or obese,^3, 6^ but not with having CVD or diabetes. None of the health outcomes of frequent fast food and quick service use were associated with living close to such premises after adjusting for demographics and socioeconomic status. These results confirmed earlier findings that proximity to fast food and quick service restaurants did not seem to affect health.^17, 18, 19^

In this study, the respondents' food environment extended beyond that of both the home and the work neighborhoods: for the ⩾2 times per week users, the median network travel distance to the fast food and quick service restaurant that they reported using was 3.82 miles away from their home and 2.20 miles away from their work place. This was almost five times longer than the median distance to nearest such restaurant to their home (0.79 mile); and almost 16 times that same distance to the nearest such restaurant to their work (0.14 mile). In research done in Atlanta, GA, the trip to a fast food restaurant was similar, with a mean of 4.96 miles (s.d. 4.41),^40^ pointing to the prevalence of car travel to these destinations.^41^ In the present study, 65% of the respondents using a fast food or quick service restaurant ⩾2 times per week reported driving to it. These results indicated that fast food users were more likely to drive to these restaurants that were further away from their home or work place.

Clearly, populations with low levels of mobility might be more influenced by the environment near their home. Children living in the lower-income neighborhoods of UK and US towns with higher density of fast food outlets were more likely to be obese.^42, 43^ For adolescents, having exposure to poor quality food environments negatively affected eating patterns and had a positive relation to weight.^44^ Proximity to fast food restaurants near home and school was also linked to a lower Healthy Eating Index.^45^ Yet many studies of children and youth also found no association between proximity to fast food restaurants and health behaviors and outcomes,^46, 47^ even though there is evidence that fast food restaurants tend to cluster near schools in low-income neighborhoods.^48^

The present findings do not rule out the possible influence of the food environment near home on out-of-home eating habits. Whatever the level of mobility, a person's choice of an eating establishment might still be indirectly related to exposure to such restaurants near home, as, for example, to live close to such restaurants might reinforce the decision to eat at similar places. One recent study using travel data in Montreal, Canada, yielded mixed results considering the effects of exposure to fast foods on being overweight in both the residential and the 'non-residential' neighborhood. Future studies need to examine the complete spatial extent of food consumption habits, which includes the home- and work-based environments as well as any other environment where eating takes place.

As in many other studies, demographic and socioeconomic characteristics were strong predictors of the health outcomes examined in this study. The role of gender remains an important consideration. Frequent fast food and quick service users who were male had an increased risk of being overweight, but not being obese, with no effect of neighborhood exposure. A recent longitudinal study based on the Framingham Heart Study Offspring Cohort found that for females, associations between proximity to fast food restaurants and increased BMI were significant; but the study did not include measures of restaurant use.^49^ In the present analyses, older age moderately predicted all of the outcomes, except for being obese. Higher socio-economic status was protective of being in perceived poor health; higher education attainment and income were protective of being obese, but not of being overweight, again with no home neighborhood exposure effect. At odds with the present study, a recent longitudinal study found that for low-income populations, proximity to fast food restaurants was associated with higher fast food consumption.^50^ Again, however, no measure of restaurant use was available.

This study's strengths lied in the availability of data about where people ate away from home, and in the individual-level analyses of behavior and environment. While environment was measured objectively, however, behavior and health outcomes were self-reported, thus subject to possible bias.^51^ The individual-level analyses bypass the limitations of area-based measures used in other studies,^52^ but the cross-sectional design means that the direction of associations is unknown.

Finally, the study comprised a sample of King County's adult population. Populations living in areas that have a different distribution of health outcomes might yield different results. Also the number and spatial distribution of fast food restaurants may vary by region, thus affecting measures of exposure and access.^53^

## Conclusions

Eating ⩾2 times a week at a fast food or quick service restaurant is associated with perceived poor health, added weight and obesity. However, living close to such restaurants is not related to negative health outcomes. Eating out at these restaurants takes place beyond the home neighborhood. To further examine whether exposure and access to fast food and quick service restaurants influence eating out behaviors, studies will need to consider the complete spatial extent of food consumption habits.

## Figures and Tables

**Table 1:** Summary statistics of samples

*Characteristic*	*Total population count (% within category) or mean (s.d.)*	*<2 per week count (% within category) or mean (s.d.)*	⩾*2 per week count (% within category) or mean (s.d.)*
*N*	2001^a^	1584	408
*Gender*			
Male	766 (38%)	566 (36%)	199 (49%)
Female	1235 (62%)	1018 (64%)	209 (51%)
Age (years)	54 (15)	56 (15)	50 (13)
Household size (persons)	2.32 (1.4)	2.3 (1.5)	2.4 (1.4)
White, non-Hispanic	1561 (78%)	1250 (79%)	315 (78%)
Number of children (<12 years)	0.32 (0.8)	0.31 (0.8)	0.37 (0.8)
Number of children (12–18 years)	0.22 (0.6)	0.2 (0.6)	0.24 (0.6)
Employment (employed)	1213 (61%)	913 (58%)	299 (74%)
Education (>some college)	1618 (82%)	1276 (81%)	336 (83%)
*Income* ($)			
<50 000	692 (40%)	556 (41%)	131 (35%)
50 000–100 000	602 (35%)	480 (35%)	122 (34%)
>100 000	446 (25%)	333 (24%)	111 (31%)
*Distance*			
Distance home to closest fast food restaurant (mile)	0.81 (0.82), Median: 0.81	1.02 (0.86), Median: 0.80	0.99 (0.88), Median: 0.83
Distance home to closest quick service restaurant (mile)	1.02 (0.87), Median: 0.60	0.82 (0.83), Median: 0.60	0.78 (0.79), Median: 0.62
Distance home to used fast food/quick service restaurant (mile)	NA^c^	NA^c^	6.0 (6.4)^b^, Median: 3.82
Distance work to used fast food/quick service restaurant (mile)	NA^c^	NA^c^	5.3 (7.4)^b^, Median: 2.20
			
*Health*			
Poor health (yes)	273 (14%)	205 (13%)	67 (17%)
BMI	26.5 (5.5)	26.2 (5.3)	27.8 (6.0)
Overweight (yes)	1041 (56%)	791 (53%)	247 (65%)
Obese (yes)	385 (21%)	271 (18%)	112 (29%)
CVD (yes)	194 (10%)	161 (10%)	32 (8%)
Diabetes (yes)	184 (9%)	146 (9%)	37 (9%)

Abbreviations: BMI, body mass index; CVD, cardiovascular disease.

a Nine respondents did not report on fast food or quick service use.

b Of the 408 participants eating ⩾2 times per week at a fast food or quick service restaurant, 347 reported the location of their primary restaurant. Among them, 40 used the closest to their home (respondents were considered to eat at the closest fast food or quick service restaurant if the network distance difference between the two was ⩽0.25 mile).

c Among all respondents, 1055 did not report using a quick service or fast food restaurant.

**Table 2:** Model results of reporting poor health for fast food or quick service restaurant use

*Variable*	*Value*	*Model 1*			*Model 2*			*Model 3*			*Model 4*		
		*OR*	*95% CI*		*OR*	*95% CI*		*OR*	*95% CI*		*OR*	*95% CI*	
⩾2 per week use of fast food or quick service	No	1.00			1.00			1.00			1.00		
	Yes	1.32	0.98	1.78	1.32	0.97	1.78	**1.38**	**1.01**	**1.90**	**1.61**	**1.13**	**2.28**
Distance to closest quick service restaurant					**0.76**	**0.61**	**0.93**	**0.79**	**0.64**	**0.98**	0.83	0.66	1.05
Age								**1.01**	**1.00**	**1.02**	1.00	0.99	1.01
Gender	Female							1.00			1.00		
	Male							1.13	0.86	1.47	1.18	0.88	1.60
White	No							1.00			1.00		
	Yes							**0.65**	**0.47**	**0.89**	0.75	0.53	1.08
Number of children under 12								0.96	0.70	1.30	0.82	0.59	1.13
Number of children of 12–18								1.07	0.74	1.53	1.01	0.68	1.49
Household size								0.90	0.74	1.09	1.04	0.85	1.27
Employment	No										1.00		
	Yes										**0.53**	**0.38**	**0.74**
Education	No college										1.00		
	College										**0.57**	**0.41**	**0.80**
Income	<$50 000										1.00		
	$50 000–100 000										**0.51**	**0.35**	**0.73**
	>$100 000										**0.32**	**0.19**	**0.52**

Abbreviations: CI, confidence interval; OR, odds ratio. Bold numbers indicate significant at 0.05 level.

**Table 3:** Model results of being overweight, obese, having a cardiovascular disease (CVD) or diabetes for fast food or quick service restaurant use

*Variable*	*Value*	*Model 5 (overweight)*			*Model 6 (obese)*			*Model 7 (CVD)*			*Model 8 (diabetes)*		
		*OR*	*95%* *CI*		*OR*	*95%* *CI*		*OR*	*95%* *CI*		*OR*	*95%* *CI*	
⩾2 per week use of fast food	No	1.00			1.00			1.00			1.00		
	Yes	**1.66**	**1.28**	**2.16**	**2.02**	**1.52**	**2.69**	0.95	0.59	1.53	1.29	0.83	1.99
Distance to closest fast food restaurant		1.14	0.99	1.30	1.12	0.96	1.31	0.83	0.64	1.09	1.03	0.82	1.28
Age		**1.01**	**1.00**	**1.02**	1.00	0.99	1.01	**1.05**	**1.03**	**1.06**	**1.02**	**1.01**	**1.04**
Gender	Female	1.00			1.00			1.00			1.00		
	Male	**2.23**	**1.80**	**2.75**	1.13	0.88	1.46	**1.91**	**1.34**	**2.73**	1.21	0.85	1.73
White	No	1.00			1.00			1.00			1.00		
	Yes	0.88	0.67	1.15	1.36	0.97	1.91	1.05	0.66	1.67	0.69	0.45	1.04
Number of children under 12		0.96	0.78	1.18	0.80	0.62	1.04	0.76	0.46	1.23	0.91	0.61	1.35
Number of children of 12–18		0.86	0.67	1.11	0.84	0.61	1.15	0.74	0.42	1.31	1.24	0.79	1.94
Household size		1.08	0.94	1.25	1.15	0.98	1.36	1.17	0.90	1.51	1.01	0.79	1.28
Employment	No	1.00			1.00			1.00			1.00		
	Yes	1.13	0.89	1.43	1.32	0.98	1.77	**0.47**	**0.31**	**0.72**	**0.51**	**0.34**	**0.77**
Education	No college	1.00			1.00			1.00			1.00		
	College	0.82	0.62	1.09	**0.71**	**0.52**	**0.97**	0.98	0.64	1.49	0.69	0.47	1.04
Income	<$50,000	1.00			1.00			1.00			1.00		
	$50,000-100,000	1.07	0.84	1.39	**0.60**	**0.45**	**0.81**	0.79	0.51	1.21	0.90	0.60	1.37
	>$100,000	0.77	0.57	1.03	**0.36**	**0.25**	**0.52**	0.64	0.37	1.12	0.59	0.33	1.04

Abbreviations: CI, confidence interval; OR, odds ratio. Bold numbers indicate significant at 0.05 level.

## Notes

Dr Drewnowski advises McDonald's Corporation on global issues related to public health nutrition.

## References

[bib1] Wang MC Cubbin C Ahn D Winkleby MA Changes in neighbourhood food store environment, food behaviour and body mass index, 1981-1990 Public Health Nut 2008 11 963 970 10.1017/S136898000700105X PMC8843036 17894915

[bib2] Nielsen SJ Siega-Riz AM Popkin BM Trends in energy intake in U.S. between 1977 and 1996: similar shifts seen across age groups Obes Res 2002 10 370 378 12006636 10.1038/oby.2002.51

[bib3] Duffey KJ Gordon-Larsen P Steffen LM Jacobs DR Jr. Popkin BM Regular consumption from fast food establishments relative to other restaurants is differentially associated with metabolic outcomes in young adults J Nutr 2009 139 2113 2118 19776183 10.3945/jn.109.109520 PMC2762152

[bib4] Moore LV Diez Roux AV Nettleton JA Jacobs DR Franco M Fast-food consumption, diet quality, and neighborhood exposure to fast food: the multi-ethnic study of atherosclerosis Am J Epidemiol 2009 170 29 36 19429879 10.1093/aje/kwp090 PMC2733038

[bib5] Fowles ER Timmerman GM Bryant M Kim S Eating at fast-food restaurants and dietary quality in low-income pregnant women West J Nurs Res 2011 33 630 651 21131508 10.1177/0193945910389083 PMC13398072

[bib6] Casey AA Elliott M Glanz K Haire-Joshu D Lovegreen SL Saelens BE Impact of the food environment and physical activity environment on behaviors and weight status in rural U.S. communities Prev Med 2008 47 600 604 18976684 10.1016/j.ypmed.2008.10.001 PMC2650016

[bib7] Morse KL Driskell JA Observed sex differences in fast-food consumption and nutrition self-assessments and beliefs of college students Nutr Res 2009 29 173 179 19358931 10.1016/j.nutres.2009.02.004

[bib8] Nielsen SJ Siega-Riz AM Popkin BM Trends in food locations and sources among adolescents and young adults Prev Med 2002 35 107 113 12200094 10.1006/pmed.2002.1037

[bib9] Fleischhacker SE Evenson KR Rodriguez DA Ammerman AS A systematic review of fast food access studies Obes Rev 2011 10.1111/j.1467-789X.2010.00715.x 20149118

[bib10] Larson N Story M A review of environmental influences on food choices Ann Behav Med 2009 38 S56 S73 19802648 10.1007/s12160-009-9120-9

[bib11] Morland K Wing S Diez Roux A The contextual effect of the local food environment on residents' diets: the atherosclerosis risk in communities study Am J Public Health 2002 92 1761 1767 12406805 10.2105/ajph.92.11.1761 PMC1447325

[bib12] Morland KB Evenson KR Obesity prevalence and the local food environment Health Place 2009 15 491 495 19022700 10.1016/j.healthplace.2008.09.004 PMC4964264

[bib13] Zick CD Smith KR Fan JX Brown BB Yamada I Kowaleski-Jones L Running to the store? The relationship between neighborhood environments and the risk of obesity Soc Sci Med 2009 69 1493 1500 19766372 10.1016/j.socscimed.2009.08.032 PMC2791711

[bib14] Maddock J The relationship between obesity and the prevalence of fast food restaurants: state-level analysis Am J Health Promot 2004 19 137 143 15559714 10.4278/0890-1171-19.2.137

[bib15] Bodor J Rice J Farley T Swalm C Rose D The association between obesity and urban food environments J Urban Health 2010 87 771 781 20458548 10.1007/s11524-010-9460-6 PMC2937132

[bib16] Inagami S Cohen DA Brown AF Asch SM Body mass index, neighborhood fast food and restaurant concentration, and car ownership J Urban Health 2009 86 683 695 19533365 10.1007/s11524-009-9379-y PMC2729867

[bib17] Hickson DA Diez Roux AV Smith AE Tucker KL Gore LD Zhang L Associations of fast food restaurant availability with dietary intake and weight among African Americans in the Jackson Heart Study, 2000-2004 Am J Public Health 2011 101 S301 S309 21551382 10.2105/AJPH.2010.300006 PMC3222494

[bib18] Pearce J Hiscock R Blakely T Witten K A national study of the association between neighbourhood access to fast-food outlets and the diet and weight of local residents Health Place 2009 15 193 197 18499502 10.1016/j.healthplace.2008.04.003

[bib19] Crawford DA Timperio AF Salmon JA Baur L Giles-Corti B Roberts RJ Neighbourhood fast food outlets and obesity in children and adults: the CLAN Study Int J Pediatr Obes 2008 3 249 256 18608630 10.1080/17477160802113225

[bib20] Rundle A Neckerman KM Freeman L Lovasi GS Purciel M Quinn J Neighborhood food environment and walkability predict obesity in New York City Environ Health Perspect 2009 117 442 447 19337520 10.1289/ehp.11590 PMC2661915

[bib21] Li F Harmer P Cardinal BJ Bosworth M Johnson-Shelton D Moore JM Built environment and 1-year change in weight and waist circumference in middle-aged and older adults: Portland Neighborhood Environment and Health Study Am J Epidemiol 2009 169 401 408 19153214 10.1093/aje/kwn398 PMC2726645

[bib22] Daniel M Paquet C Auger N Zang G Kestens Y Association of fast-food restaurant and fruit and vegetable store densities with cardiovascular mortality in a metropolitan population Eur J Epidemiol 2010 25 711 719 20821254 10.1007/s10654-010-9499-4

[bib23] Morgenstern LB Escobar JD Sanchez BN Hughes R Zuniga BG Garcia N Fast food and neighborhood stroke risk Ann Neurol 2009 66 165 170 19743456 10.1002/ana.21726 PMC2745509

[bib24] Azuma AM Gilliland S Vallianatos M Gottlieb R Food access, availability, and affordability in 3 Los Angeles communities, Project CAFE, 2004-2006 Prev Chronic Dis 2010 7 A27 20158956 PMC2831781

[bib25] Lovasi GS Hutson MA Guerra M Neckerman KM Built environments and obesity in disadvantaged populations Epidemiol Rev 2009 31 7 20 19589839 10.1093/epirev/mxp005

[bib26] Fraser LK Edwards KL Cade J Clarke GP The geography of Fast Food outlets: a review Int J Environ Res Public Health 2010 7 2290 2308 20623025 10.3390/ijerph7052290 PMC2898050

[bib27] Hemphill E Raine K Spence JC Smoyer-Tomic KE Exploring obesogenic food environments in Edmonton, Canada: the association between socioeconomic factors and fast-food outlet access Am J Health Promot 2008 22 426 432 18677883 10.4278/ajhp.22.6.426

[bib28] Smoyer-Tomic KE Spence JC Raine KD Amrhein C Cameron N Yasenovskiy V The association between neighborhood socioeconomic status and exposure to supermarkets and fast food outlets Health Place 2008 14 740 754 18234537 10.1016/j.healthplace.2007.12.001

[bib29] Black JL Macinko J Dixon LB Fryer JGE Neighborhoods and obesity in New York City Health Place 2010 16 489 499 20106710 10.1016/j.healthplace.2009.12.007

[bib30] Jones J Terashima M Rainham D Fast food and deprivation in Nova Scotia Can J Public Health 2009 100 32 35 19263973 10.1007/BF03405489 PMC6973878

[bib31] Spence JC Cutumisu N Edwards J Raine KD Smoyer-Tomic K Relation between local food environments and obesity among adults BMC Public Health 2009 9 192 19538709 10.1186/1471-2458-9-192 PMC2708156

[bib32] Hurvitz PM Moudon AV Rehm CD Streichert LC Drewnowski A Arterial roads and area socioeconomic status are predictors of fast food restaurant density in King County, WA Int J Behav Nutr Phys Act 2009 6 46 19630979 10.1186/1479-5868-6-46 PMC2724491

[bib33] Sharkey JR Johnson CM Dean WR Horel SA Association between proximity to and coverage of traditional fast-food restaurants and non-traditional fast-food outlets and fast-food consumption among rural adults Int J Health Geogr 2011 10 37 21599955 10.1186/1476-072X-10-37 PMC3112378

[bib34] Timperio AF Ball K Roberts R Andrianopoulos N Crawford DA Children's takeaway and fast-food intakes: associations with the neighbourhood food environment Public Health Nutr 2009 12 1960 1964 19243674 10.1017/S1368980009004959

[bib35] Pereira MA Kartashov AI Ebbeling CB Van Horn L Slattery ML Jacobs JDR Fast-food habits, weight gain, and insulin resistance (the CARDIA study): 15-year prospective analysis Lancet 2005 365 36 42 15639678 10.1016/S0140-6736(04)17663-0

[bib36] Simon PA Kwan D Angelescu A Shih M Fielding JE Proximity of fast food restaurants to schools: Do neighborhood income and type of school matter Prev Med 2008 47 284 288 18448158 10.1016/j.ypmed.2008.02.021

[bib37] Wang MC Gonzalez AA Ritchie LD Winkleby MA The neighborhood food environment: sources of historical data on retail food stores Int J Behav Nutr Phys Act 2006 3 15 16846518 10.1186/1479-5868-3-15 PMC1564032

[bib38] US Census Bureau. North American Industry Classification System (NAICS) Definitions, Food and Beverage Stores. 2007. Available from http://www.census.gov/cgi-bin/sssd/naics/naicsrch?code=445&search=2007%20NAICS%20Search (Accessed 24 August 2011).

[bib39] Vernez Moudon A Drewnowski A Duncan GE Hurvitz PM Saelens BE Scharnhorst E Characterizing the food environment: pitfalls and future directions Public Health Nutr 2013 16 1238 1243 23570695 10.1017/S1368980013000773 PMC3975130

[bib40] Kerr J Frank L Sallis JF Saelens B Glanz K Chapman J Predictors of trips to food destinations Int J Behav Nutr Phys Act 2012 9 58 22607218 10.1186/1479-5868-9-58 PMC3413585

[bib41] Thornton LE Crawford DA Cleland VJ Timperio AF Abbott G Ball K Do food and physical activity environments vary between disadvantaged urban and rural areas? Findings from the READI Study Health Promot J Austr 2012 23 153 156 23088479 10.1071/he12153

[bib42] Frazier DA The link between fast food and the obesity epidemic Health Matrix Clevel 2007 17 291 317 18326394

[bib43] Oreskovic NM Winickoff JP Kuhlthau KA Romm D Perrin JM Obesity and the built environment among Massachusetts children Clin Pediatr (Phila) 2009 48 904 912 19487763 10.1177/0009922809336073

[bib44] Davis B Carpenter C Proximity of fast-food restaurants to schools and adolescent obesity Am J Public Health 2009 99 505 510 19106421 10.2105/AJPH.2008.137638 PMC2661452

[bib45] He M Tucker P Irwin JD Gilliland J Larsen K Hess P Obesogenic neighbourhoods: the impact of neighbourhood restaurants and convenience stores on adolescents' food consumption behaviours Public Health Nutr 2012 15 2331 2339 22390896 10.1017/S1368980012000584 PMC10271768

[bib46] Richardson AS Boone-Heinonen J Popkin BM Gordon-Larsen P Neighborhood fast food restaurants and fast food consumption: a national study BMC Public Health 2011 11 543 21740571 10.1186/1471-2458-11-543 PMC3160374

[bib47] Powell LM Fast food costs and adolescent body mass index: evidence from panel data J Health Econ 2009 28 963 970 19732982 10.1016/j.jhealeco.2009.06.009

[bib48] Kestens Y Daniel M Social inequalities in food exposure around schools in an urban area Am J Prev Med 2010 39 33 40 20537844 10.1016/j.amepre.2010.03.014

[bib49] Block JP Christakis NA O'Malley AJ Subramanian SV Proximity to food establishments and body mass index in the Framingham Heart Study offspring cohort over 30 years Am J Epidemiol 2011 174 1108 1114 21965186 10.1093/aje/kwr244 PMC3208142

[bib50] Boone-Heinonen J Gordon-Larsen P Kiefe CI Shikany JM Lewis CE Popkin BM Fast food restaurants and food stores: longitudinal associations with diet in young to middle-aged adults: the CARDIA study Arch Intern Med 2011 171 1162 1170 21747011 10.1001/archinternmed.2011.283 PMC3178268

[bib51] Idler E Benyamini Y Self-rated health and mortality: a review of twenty-seven community studies Journal of Health and Social Behavior 1997 38 21 37 9097506

[bib52] Feng J Glass TA Curriero FC Stewart WF Schwartz BS The built environment and obesity: a systematic review of the epidemiologic evidence Health Place 2010 16 175 190 19880341 10.1016/j.healthplace.2009.09.008

[bib53] Raja S Ma C Yadav P Beyond food deserts: measuring and mapping racial disparities in neighborhood food environments J Planning Educ Res 2008 27 469 482

